# Identifying Cancers Impacted by CDK8/19

**DOI:** 10.3390/cells8080821

**Published:** 2019-08-03

**Authors:** Igor B. Roninson, Balázs Győrffy, Zachary T. Mack, Alexander A. Shtil, Michael S. Shtutman, Mengqian Chen, Eugenia V. Broude

**Affiliations:** 1 Department of Drug Discovery and Biomedical Sciences, University of South Carolina, Columbia, SC 29208, USA; 2 Institute of Gene Biology, Russian Academy of Sciences, 119334 Moscow, Russia; 3 MTA TTK Lendület Cancer Biomarker Research Group, Institute of Enzymology, Hungarian Academy of Sciences, Magyar tudósok körútja 2., H-1117 Budapest, Hungary; 4 Department of Pediatrics, Semmelweis University, Tűzoltó utca 7-9, H-1094 Budapest, Hungary; 5 Blokhin National Medical Research Center of Oncology, 115478 Moscow, Russia

**Keywords:** CDK8, CDK19, Cyclin C, cancer genomics, survival correlations

## Abstract

CDK8 and CDK19 Mediator kinases are transcriptional co-regulators implicated in several types of cancer. Small-molecule CDK8/19 inhibitors have recently entered or are entering clinical trials, starting with breast cancer and acute myeloid leukemia (AML). To identify other cancers where these novel drugs may provide benefit, we queried genomic and transcriptomic databases for potential impact of CDK8, CDK19, or their binding partner CCNC. sgRNA analysis of a panel of tumor cell lines showed that most tumor types represented in the panel, except for some central nervous system tumors, were not dependent on these genes. In contrast, analysis of clinical samples for alterations in these genes revealed a high frequency of gene amplification in two highly aggressive subtypes of prostate cancer and in some cancers of the GI tract, breast, bladder, and sarcomas. Analysis of survival correlations identified a group of cancers where CDK8 expression correlated with shorter survival (notably breast, prostate, cervical cancers, and esophageal adenocarcinoma). In some cancers (AML, melanoma, ovarian, and others), such correlations were limited to samples with a below-median tumor mutation burden. These results suggest that Mediator kinases are especially important in cancers that are driven primarily by transcriptional rather than mutational changes and warrant an investigation of their role in additional cancer types.

## 1. Introduction

The Mediator kinase CDK8 and its paralog CDK19, together with their binding partner Cyclin C (CCNC), have been implicated through experimental studies in several malignancies, including cancers of the colon [1,2,3], breast [4,5,6,7], prostate [8], pancreas [9], melanoma [10], and leukemias [11,12]. A number of small-molecule CDK8/19 inhibitors have been developed [13]. Such inhibitors showed potentially beneficial effects on leukemia cell proliferation [11,12], tumor invasion [9,14], metastasis [3], or tumor response to other anti-cancer drugs [4,6]. These effects of CDK8/19 inhibitors have been demonstrated in several in vivo models, including lung [4], breast [6], colon [3], prostate cancers [15], and leukemia [11]. The first clinical trials of Mediator kinase inhibitors have been conducted in estrogen receptor–positive breast cancers (ClinicalTrials.gov Identifier: NCT03065010) and should be initiated soon in acute myeloid leukemia (AML). It is now becoming imperative to identify other types of cancer and categories of patients who could benefit from this new class of drugs. 

Unlike better-known member of the cyclin-dependent kinase (CDK) family—such as CDK1, CDK2, and CDK4/6—CDK8/19 do not mediate cell cycle progression but are only involved in transcription [16,17]. Furthermore, in contrast to the best-known transcriptional CDKs, such as CDK7 and CDK9, CDK8/19 activity is not required for transcription in general and does not affect normal cell growth. Instead, CDK8/19 regulate transcription in a selective gene-specific context, primarily enhancing the induction of silent genes as they become activated by various transcription factors [18]. Since CDK8/19 regulate transcription but not cell cycle progression, very few cancers, primarily a subset of leukemias [11], were found to require CDK8/19 for their proliferation. On the other hand, selective CDK8/19 inhibitors showed a variety of therapeutic activities in vivo that are not based on the inhibition of tumor cell proliferation, such as suppression of tumor-promoting effects of chemotherapy-induced DNA damage of host tissues [4], selective inhibition of metastatic but not primary growth of colon cancers [3], and potentiation of in vivo effects of doxorubicin [4] or fulvestrant [6]. With this emerging picture, it seems unlikely that cancers where CDK8/19 inhibitors would have a beneficial impact will be defined as those that are growth-suppressed in vitro by CDK8/19 inhibition. On the other hand, potential target cancers could be identified through informatic analysis of clinical data, which may reveal tumor types where CDK8/19 or their binding partner Cyclin C (CCNC) would be amplified or overexpressed or where CDK8/19 expression would be associated with bad prognosis. In the present study, we have analyzed the available genomic and transcriptomic databases to identify cancers where Mediator kinases are likely to play essential roles.

## 2. Materials and Methods

DepMap (https://depmap.org/portal/) analysis of the dependency of a panel of tumor cell lines on individual genes [19,20] was conducted using the most recent (May 2019) CRISPR (Avana) Public 19Q2 and Combines RNAi (Broad, Novartis, Marcotte) databases. 

cBioPortal for cancer genomics (https://www.cbioportal.org/) was used to query the alterations of CDK8/CDK19/CCNC in clinical tumor samples at the DNA level and gene expression at the RNA level. To maximize the likelihood of discovery of the alterations, DNA analysis was carried out using a set of 159 transcriptomic studies (TCGA- and non-TCGA) that were manually curated with no overlapping samples (40,199 samples total). The TCGA dataset was used to query gene expression in different cancers at the RNA level. 

SurvExpress (http://bioinformatica.mty.itesm.mx:8080/Biomatec/SurvivaX.jsp) was used to analyze gene expression in three equally sized risk groups in the curated SurvExpress dataset of microarray data in 808 colon cancers.

For most of the tumor types the survival analysis was carried out using the Pan-Cancer datasets of the www.kmplot.com online tool [21]. The Pan-Cancer dataset is based on the TCGA data generated using the Illumina HiSeq 2000 platform with survival information derived from a previous publication [22]. In the survival analysis, each cutoff between the lower and upper quartiles was analyzed by Cox proportional hazards regression and the best performing cutoff was used in the final analysis. Kaplan-Meier survival plots were derived to visualize the survival curves, and hazard rates were computed to numerically depict the difference between the two cohorts. The analyses were performed using the “survival” package in the R statistical environment (http://www.r-project.org). The final plots were formatted in Microsoft Excel using the WinStat 2019 extension (R Fitch Software, Cambridge, MA, USA).

Tumor mutation burden (TMB) was determined for each sample by computing the total number of mutated genes using the whole exome-sequencing data of the tumors. Genes with multiple mutations were counted only once. The median number of mutations within the exome was computed separately for each tumor type. Samples with higher mutation count were designated as TMB-high and those with a lower mutation count as TMB-low. The correlation between survival and CDK8 expression was analyzed in all samples combined and separately in TMB-high and -low samples.

## 3. Results

### 3.1. Analysis of Tumor Cell Line Dependency on CDK8/CDK19/CCNC

For most classes of targeted drugs, an obvious approach to identifying potential target cancers would be to screen a large panel of cell lines representing different tumor types for in vitro growth inhibition by the drug. As a notable recent example, this approach was used to identify estrogen receptor (ER)-positive breast cancer as a target disease for the inhibitors of CDK4/6 kinases essential for cell cycle progression in G1 [23]. Following this analysis, CDK4/6 inhibitors underwent successful trials and were rapidly approved for this disease [24]. Today, an online approach is available to predict and approximate the results of such screening, thanks to the DepMap project conducted by the Broad Institute in collaboration with the Wellcome Sanger Institute (www.depmap.org) [19,20]. In this project, a comprehensive library of human genes has been knocked down or, more recently, knocked out through CRISPR technology in large panels of human cell lines representing different types of cancer, followed by enrichment/depletion analysis of gene-specific shRNAs or sgRNAs. The probability of dependency of each cell line on the queried gene is represented as dependency scores, where the strong negative values mark those cell lines where a given gene is especially important for growth or survival. 

**Figure 1:**
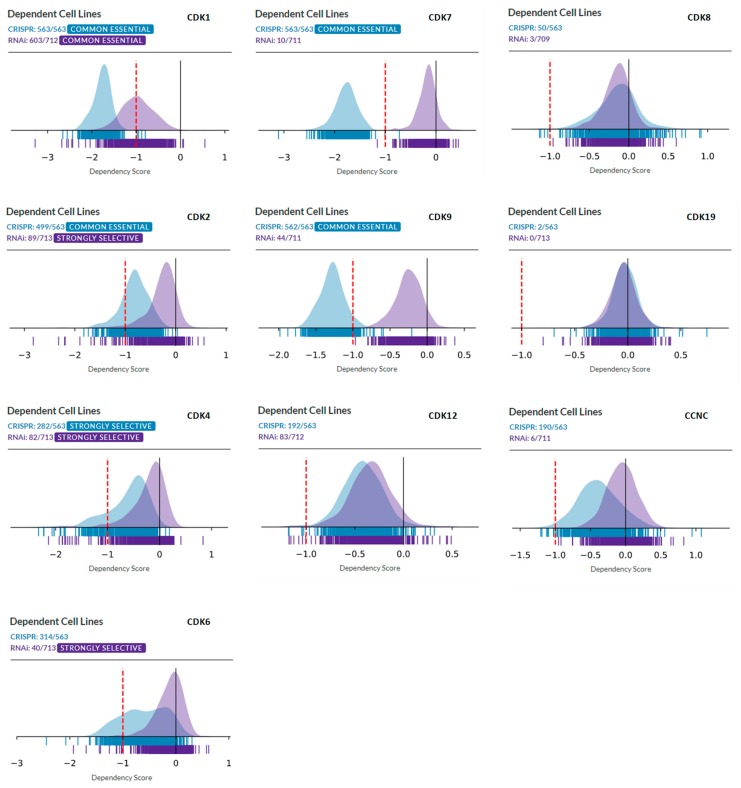
DepMap analysis of the dependency of tumor cell line panels in CRISPR (blue) and RNAi (violet) databases on the indicated CDKs. *X*-axis: dependency scores. Left column: cell cycle CDKs. Middle column: transcriptional CDKs. Right column: CDK8, CDK19, and CCNC.

Figure 1 compares the overall distributions of DepMap dependency scores derived from CRISPR analysis of a panel of 563 tumor cell lines and RNAi analysis of a panel of 713 cell lines, for cell cycle CDKs (CDK1, CDK2, CDK4, and CDK6, left column), transcriptional CDKs (CDK7, CDK9, and CDK12, middle column), and CDK8, CDK19, and CCNC (right column). In most cases, as expected, CRISPR knockout produced much stronger effects than partial knockdown by RNAi. Analysis of cell cycle CDKs identified CDK1 as a “common essential” gene, with the majority of cell lines of different types dependent on CDK1 in both panels. CDK2 is classified as “common essential” by CRISPR analysis but RNAi analysis classifies it as “strongly selective” (this selectivity pertains to ovarian cancer, not shown). Strikingly, CDK4, the primary target of CDK4/6 inhibitors that showed a major clinical impact in ER-positive breast cancers, was classified as “strongly selective” in both CRISPR and RNAi panels, with the selectivity primarily associated with ER-positive cell lines, Ewing sarcoma and breast cancer (not shown). CDK6 was classified as “strongly selective” by RNAi (but not CRISPR) analysis (Figure 1), with selectivity for hematopoietic malignancies (not shown). 

CRISPR knockout of the most commonly targeted transcriptional CDKs, CDK7, and CDK9, showed drastic growth inhibition in almost all the cell lines, classifying these genes as “common essential.” On the other hand, shRNA knockdown of CDK7 and CDK9 had much weaker growth inhibitory effects, ranking these as neither “common essential” nor “strong selectivity.” Another transcriptional CDK that has garnered recent attention, CDK12, produced similar results in the CRISPR and RNAi analysis, with almost all the cell lines showing moderate dependency on CDK12, with no apparent strong selectivity (Figure 1).

**Figure 2:**
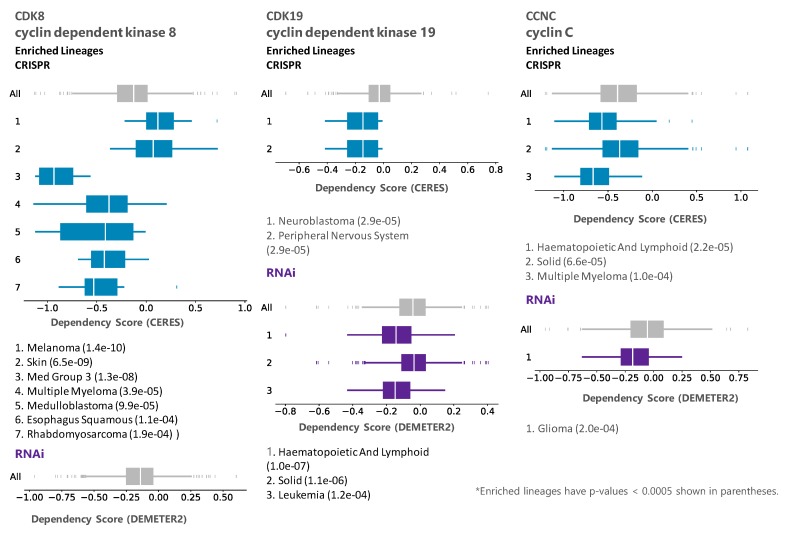
DepMap representation of lineage enrichment in the analysis of dependency of CRISPR and RNAi panels on CDK8, CDK19, and CCNC.

We then queried DepMap CRISPR and RNAi databases for dependency on CDK8, CDK19, and CCNC (Cyclin C). Remarkably, analysis of co-dependency among all the genes revealed that CDK8 showed the highest co-dependency with CCNC and vice versa (Pearson correlation 0.51) in the CRISPR database, indicating biological validity of this analysis (in the case of CDK19, no genes showed co-dependency with a Pearson correlation above 0.30). However, the knockout or knockdown of CDK8 or CDK19 showed the weakest overall growth-inhibitory activity among all the CDKs. The knockout (but not the knockdown) of CCNC, the common binding partner of both CDK8 and CDK19, and possibly CDK3, had a somewhat stronger growth inhibitory effect (Figure 1). Some tumor type selectivity was suggested by this analysis, with the strongest dependency detected for CDK8 by CRSPR assays in tumors of the central nervous system (designated Med Group 3) (Figure 2), and among the other enriched lineages, hematopoietic malignancies were identified as dependent on CDK19 by RNAi analysis (Figure 2). 

### 3.2. Analysis of CDK8/CDK19/CCNC Alterations and Expression in Clinical Cancers

**Figure 3:**
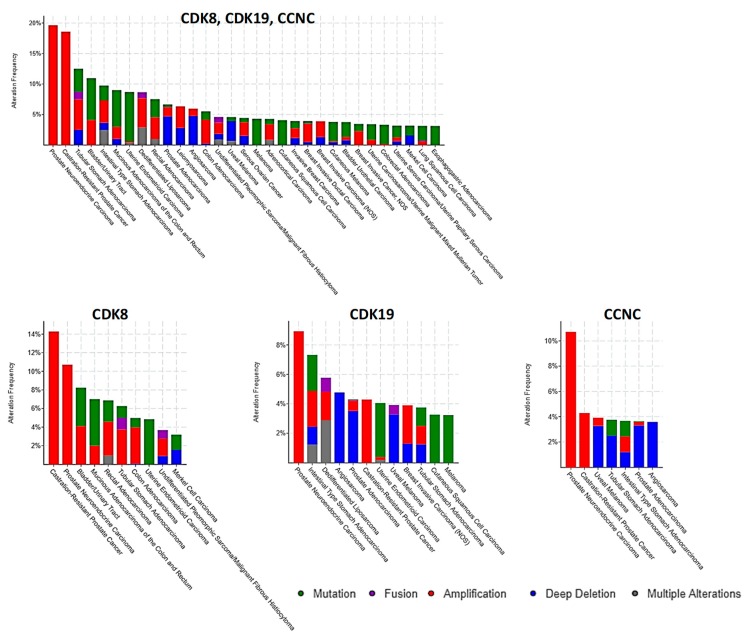
cBioPortal analysis of frequencies of the indicated alterations of CDK8, CDK19, and CCNC (gene amplification red, deep deletion blue, mutation green) among different cancer subtypes. The analysis was carried out using a set of 159 studies that were manually curated with no overlapping samples (40,199 samples total).

Our subsequent analysis concentrated on the data from clinical tumor samples rather than cell lines. Using the cBioPortal for cancer genomics (https://www.cbioportal.org/) [25], we queried the alterations of CDK8/CDK19/CCNC in clinical samples at the DNA level. This analysis was carried out using a set of 40,199 non-overlapping tumor samples. Figure 3 shows the distribution of different alterations of these three genes (gene amplification, deep deletions, mutations, fusions, multiple alterations) in various subtypes of different cancers that were represented by at least 50 samples per subtype and showed combined alteration frequencies of at least 3%. 

The greatest alteration frequency for one or more of these genes was observed in prostate neuroendocrine cancers and in castration-resistant prostate cancers (CRPC), where gene amplification was found in 19.6% (11/56) and 18.6% (13/70) samples, respectively (data from [26]). A substantial fraction (4.7%) of prostate adenocarcinomas (not further classified) showed deep deletions of one or more of these three genes, but no such deletions were found in the CRPC or neuroendocrine prostate cancer subtypes. Neuroendocrine prostate cancers and CRPC also showed the highest frequency of gene amplification for CDK8, CDK19, and CCNC individually (Figure 3). After these two subtypes of prostate cancer, gene amplification of one or more of CDK8, CDK19, and CCNC was most common in several subtypes of cancers of the GI tract (tubular stomach adenocarcinoma (5%), colon adenocarcinoma (3.9%), intestinal type stomach adenocarcinoma (3.7%), rectal adenocarcinoma (3.6%)), as well as dedifferentiated liposarcoma (4.8%), bladder/urinary tract cancer (4.11%), and breast invasive ductal carcinoma (3%) (Figure 3). 

**Figure 4:**
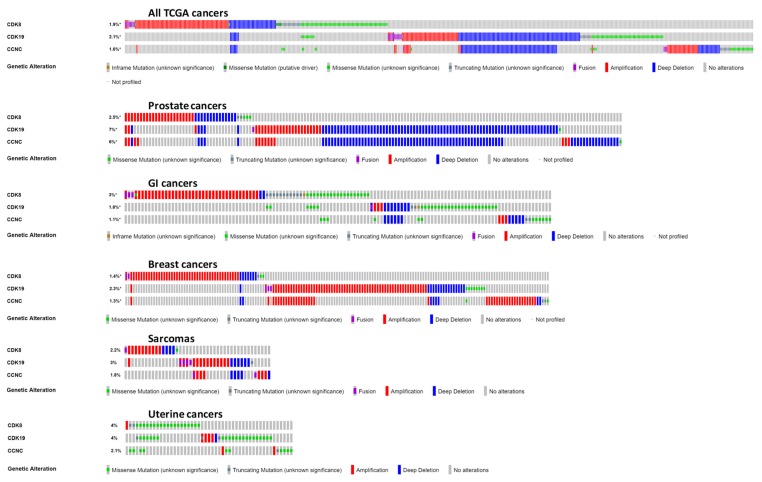
cBioPortal “oncoprint” representation of alterations in CDK8/CDK19/CCNC identified in different cancers (amplification, red; deletion, blue; mutation, green). Numbers indicate combined frequency of all alterations. All cancers in the TCGA dataset are shown above; the representation for individual cancers is based on the same selection as in Figure 3 (both TCGA- and non-TCGA).

Detailed “oncoprint” representation of CDK8/CDK19/CCNC alterations in all the types of cancer in the TCGA database [27] and in specific cancer types where the greatest numbers of alterations were found is shown in Figure 4. Examination of alterations in different cancers shows that CDK8, CDK19 and CCNC can be amplified or deleted either independently of each other or in combinations. In particular, CCNC and CDK19, both of which are located on chromosome 6 (6q16.2 and 6q21, respectively), are frequently co-amplified or co-deleted. However, there are also cases where CCNC (with or without CDK19) was co-amplified or co-deleted with CDK8, which is located on another chromosome (13q12.13), indicating combined selective pressure on the binding partners. In particular, co-amplification of CDK8 with CCNC and/or CDK19 was found in some cases of prostate and breast cancer. Among the GI tract cancers, CDK8 was amplified much more frequently than CDK19 or CCNC (Figure 4), with no amplification of CDK19 or CCNC found in colon cancers (not shown). In contrast, CDK8, CDK19, and CCNC showed similar amplification frequencies in breast cancers and sarcomas. Among other cancer types with relatively frequent alterations of CDK8, CDK19, or CCNC, we note cancers of the uterus, where mutations of these genes were found more frequently than in the other cancer types (Figure 4); most of these were missense mutations of unknown significance (Figure 4). 

**Figure 5:**
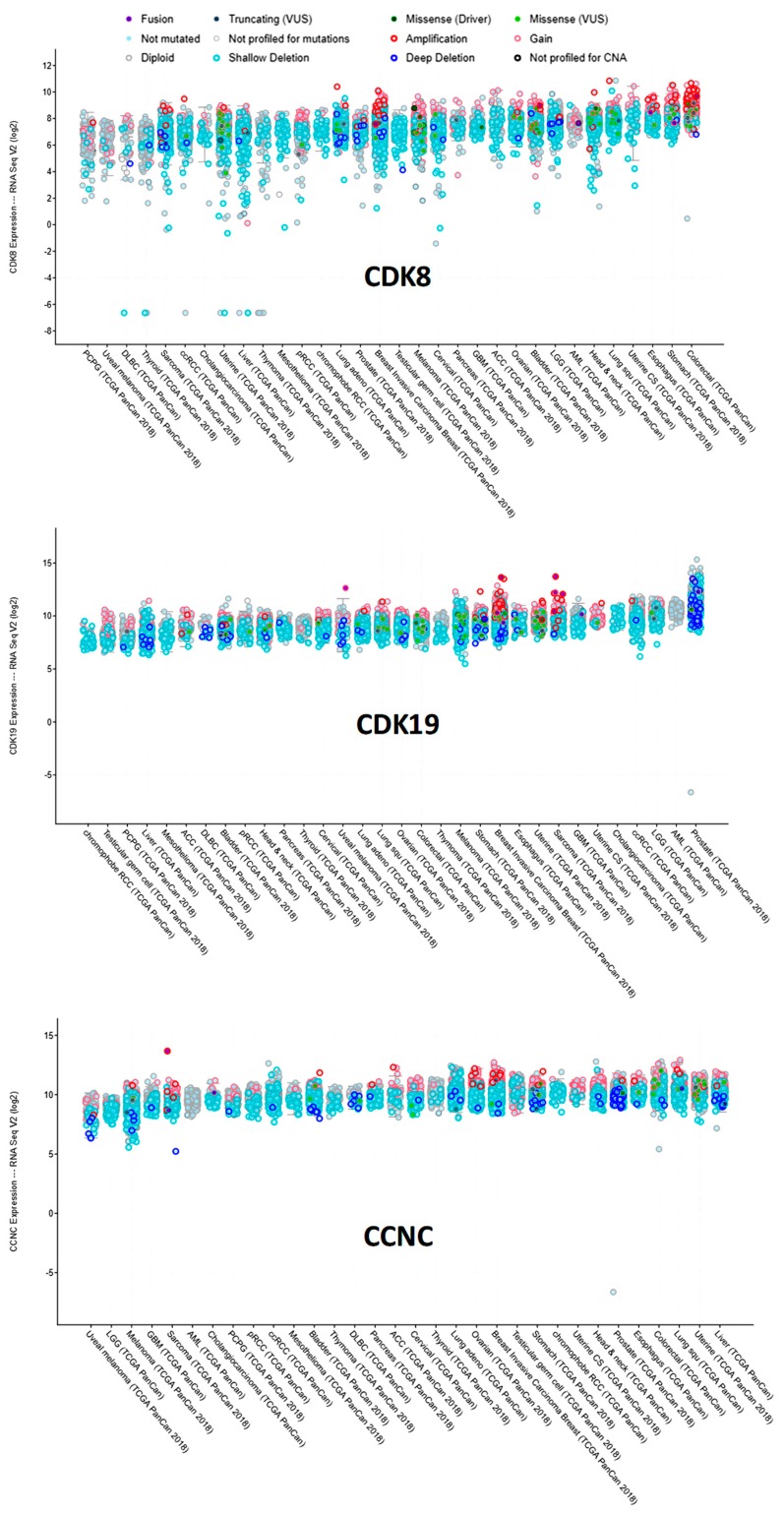
Expression of CDK8/CDK19/CCNC in different tumor types in the TCGA database (cBioPortal), arranged by median. *Y*-axis: normalized transcripts per million (TPM), log scale.

We have also used cBioPortal to query RNA-Seq data for CDK8, CDK19, and CCNC RNA expression in different tumor types in the TCGA data (Figure 5). CDK8 RNA expression was the most heterogeneous among tumors, with somewhat higher levels observed in cancers of the colon (including gene-amplified samples) and the stomach. CDK19 expression was relatively uniform, except for much higher levels in prostate cancers relative to all the others. CCNC expression was also relatively uniform, but CCNC RNA was apparently lower in melanomas (Figure 5). 

### 3.3. Analysis of Survival Correlations for CDK8/CDK19/CCNC

With this information, we investigated whether the levels of CDK8/CDK19/CCNC RNA expression in the tumors would show correlations with patient survival. We have previously carried out such analysis for breast cancer, showing that CDK8 and related genes were associated with the failure of systemic therapy in all the principal molecular subtypes of breast cancer [5]. The analysis was now carried out using RNA-Seq data in the Pan-Cancer dataset of Kaplan-Maier plotter [28] website (kmplot.com) as well as TCGA RNA-Seq data from other cancers that are not yet available at that website but have been analyzed by the same methodology. The results of this analysis for correlations of gene expression with the overall survival (OS) and relapse-free survival (RFS) (or with biochemical relapse (BCR) in the case of prostate cancer) are summarized in Table 1. The analysis was aimed at maximizing the number of cancer types that may be potentially affected by CDK8/19 and that would therefore warrant further investigation, rather than to draw definitive conclusions about the role of CDK8/19 in specific cancers. For this reason, correction for multiple hypothesis testing was omitted from the analysis. CDK8 expression in most cases showed the strongest survival correlations. CDK19 expression frequently showed similar correlations to CDK8, whereas CCNC expression often showed discordant correlations from CDK8 and CDK19 (Table 1). 

**Figure 6:**
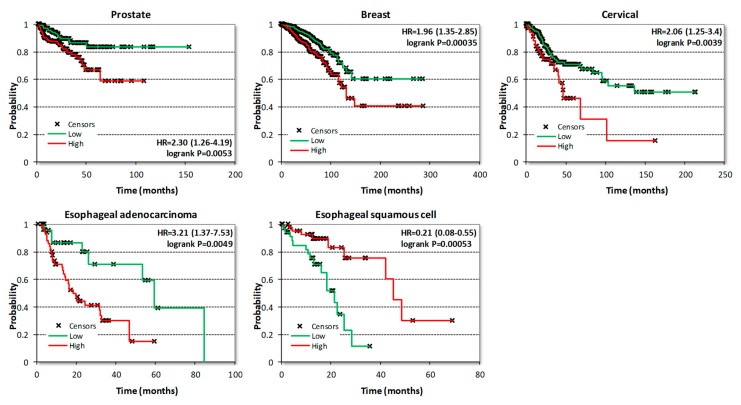
Examples of Kaplan-Meier (KM) plots correlating CDK8 expression with time to overall survival (OS) or to biochemical relapse (BCR) in the case of prostate cancer.

**Figure 7:**
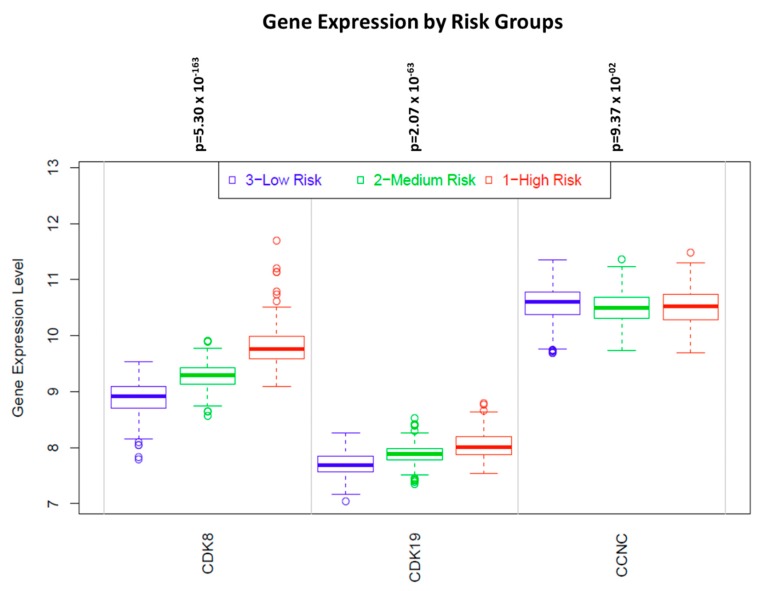
Levels of CDK8/CDK19/CCNC expression in three equal-size groups of colon cancers patients stratified by increasing risk, in the curated SurvExpress dataset (http://bioinformatica.mty.itesm.mx:8080/Biomatec/SurvivaX.jsp) of 808 colon cancers.

Figure 6 presents examples of KM plots showing some of the strongest survival correlations for CDK8 expression, including correlations with shorter BCR for prostate cancer and with shorter OS for breast and cervical cancers and esophageal adenocarcinoma. In contrast, CDK8 expression was correlated with longer OS in esophageal squamous cell carcinoma (Figure 6). Surprisingly, despite the well-documented prognostic impact of CDK8 protein in colon cancer [29], no significant correlations with OS or RFS were detected in the colon cancer RNA-Seq dataset (Table 1). On the other hand, analysis of microarray data (which is available with a longer follow-up than RNA-Seq data) in the curated SurvExpress dataset (http://bioinformatica.mty.itesm.mx:8080/Biomatec/SurvivaX.jsp) of 808 colon cancers shows that expression of CDK8 and CDK19 (but not CCNC) was progressively increased among patients separated into three risk groups according to disease-specific survival (Figure 7).

**Figure 8:**
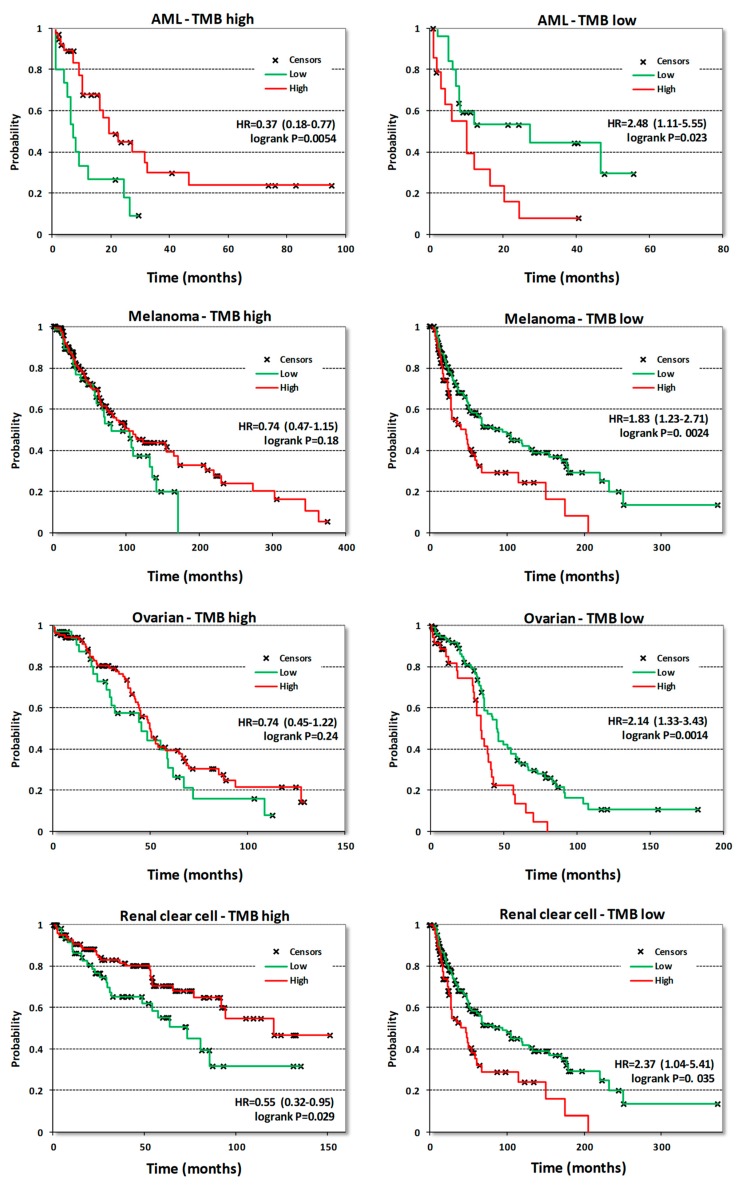
KM plots correlating CDK8 expression with OS in the indicated tumor types stratified into samples with high or low mutation burden. The high/low mutation burden cutoff for AML was 10 mutated genes per sample, for melanoma 272, for ovarian cancer 79 and for renal clear cell cancer 47.

Surprisingly, neither survival correlations nor the analysis of CDK8/CDK19/CCNC gene alterations or expression provided any rationale for using CDK8/19 inhibitors to target acute myeloid leukemia (AML), the only disease where a subset of cell lines was found to be highly susceptible to anti-proliferative effects of CDK8/19 inhibition [11,12]. In fact, CDK8/CDK19/CCNC expression in AML was correlated with longer survival (Table 1). Therefore, we have analyzed survival correlations of CDK8 expression in AML samples stratified by different criteria. One of these criteria was tumor mutation burden (TMB), determined in tumor samples that were analyzed by DNA exome sequencing. Above-median numbers of mutated genes per sample (based on the mutation frequency calculated for each tumor type) were defined as high-TMB and below-median levels as low-TMB. When AML samples were separated by this criterion, CDK8 expression was found to correlate with longer OS in samples with high TMB and with shorter OS in samples with low TMB (Figure 8). Stratification into groups with high and low TMB revealed other cancers where CDK8 expression became a marker of shorter OS only in tumors with low TMB, namely melanoma, ovarian adenocarcinoma and renal clear cell carcinoma (Figure 8). Similar correlations were seen in pancreatic cancer and sarcomas albeit the correlations with shorter OS in low-TMB samples showed p values > 0.05 (not shown). Stratification by TMB didn’t change the nature of CDK8 correlations in other cancer types.

## 4. Discussion

The analysis presented above highlights some unique aspects of Mediator kinases as cancer drug targets. In contrast to most targets of tumor-suppressive drugs (such as, for example, CDK4/6), very few tumor cell lines showed pronounced dependency on CDK8/CDK19/CCNC in DepMap analysis. Hence, the results of this analysis suggest that growth inhibition of tumor cell lines is not a very productive approach to identifying disease targets for CDK8/19 inhibitors. This conclusion agrees, in particular, with the experimental results obtained in colon cancer, where CDK8 has been identified as a frequently amplified oncogene and a negative prognostic marker [1,29] but CDK8/19 inhibitors did not suppress in vitro growth even of those cell lines where CDK8 was amplified or overexpressed. Nevertheless, small-molecule CDK8/19 inhibitors did show beneficial therapeutic effects in colon cancer models but these effects were observed only in vivo and specifically when targeting metastatic growth of colon cancers in the liver [3]. Hence, it stands to reason that the analysis of the role of the Mediator kinase in different cancers should take in account primarily in vivo correlations.

Analysis of CDK8/CDK19/CCNC alterations identified several cancer subtypes where these genes were frequently altered. The alterations included gene amplification, which is the alteration most likely to indicate a role for a gene in carcinogenesis and tumor progression, deep deletions that could be indicative of a tumor suppressor, as well as mutations in these genes. Both deletions and amplifications were especially notable among prostate cancers, where increasing expression of CDK19 and CDK8 has been shown to be associated with carcinogenesis and acquisition of the largely incurable CRPC phenotype [8]. Two treatment-resistant subtypes of prostate cancer, CRPC and neuroendocrine prostate cancers, showed the highest (close to 20%) amplification frequencies of one or more of these genes. On the other hand, a substantial fraction (close to 5%) of prostate cancers that did not belong to these two subtypes showed deep deletions of CDK8/CDK19/CCNC, suggesting that such deletions may indicate a particular subtype of prostate cancers where the Mediator kinase plays the opposite role to its carcinogenesis-associated effects in the majority of these tumors.

Other cancers with a relatively high frequency of gene amplification included GI cancers, among which colon cancers, in particular, showed a unique specificity of gene amplification for CDK8 over CDK19, in agreement with the original report [1]. The other cancers with a substantial frequency of CDK8/CDK19/CCNC gene amplification were breast cancers and sarcomas. On the other hand, uterine cancers were unique in showing a high frequency of point mutations in CDK8/CDK19/CCNC. Notably, the latter cancers are also characterized by frequent mutations of MED12 [30], a protein that interacts with the Mediator kinase in the CDK module of the Mediator, although MED12 also has Mediator-independent functions in the cytoplasm [31]. The impact of the CDK8/CDK19/CCNC mutations found in uterine cancers is unknown, but we note that uterine cancers were also one of the types that showed correlations between the expression of CDK8 and CDK19 and shorter survival.

Analysis of TCGA RNA expression data for these three genes showed that CDK8 expression was the most variable, including cases with an apparent silencing of this gene. In contrast, CDK19 RNA expression was relatively uniform among different cancers, with a prominent elevation in prostate cancers, where CDK19 has been already identified as a marker of carcinogenesis and tumor progression [8]. Interestingly, the RNA levels of CDK19 were generally much higher than those of CDK8. In contrast, at the protein level, CDK19 expression in tumor cell lines (other than prostate cancer) is generally much lower than the expression of CDK8 (our unpublished data), suggesting post-transcriptional regulation of CDK19.

Given the high range of CDK8 expression in tumor samples, it is not surprising that CDK8 RNA levels also showed the best correlations with decreased survival in several tumor types. In addition to confirming the previously reported survival correlations for breast and prostate cancers [6,8], several new tumor types showed a correlation between higher CDK8 levels and shorter survival, suggesting that these cancers could be potential targets for CDK8/19 inhibitor therapy. These include cervical cancer, esophageal adenocarcinoma, lung adenocarcinoma, liver hepatocellular carcinoma, head and neck squamous cell carcinoma, uterine endometrial carcinoma, and sarcomas. Interestingly, colon cancers for which CDK8 correlations with shorter survival have been reported at the protein level [8] did not show such correlations in the RNA-Seq database, although CDK8 expression was associated with higher risk in the microarray database, where longer follow-up times were available for survival correlations. In addition to this difference between microarray and RNA-Seq databases, regulation of CDK8 expression at protein level in colon cancers cannot be ruled out.

Remarkably, some cancers, in particular stomach adenocarcinoma and esophageal squamous cell carcinoma, showed the opposite correlations for CDK8 expression, as it was associated with longer patient survival, suggesting that the Mediator kinase may play a tumor-suppressive role in such cancers. It was especially surprising that CDK8 and CDK19 expression showed correlations (albeit weak) with a longer rather than shorter survival in AML, the only disease where a large subset of cell lines was found to be highly susceptible in vitro to CDK8/19 inhibitors [11,12]. We have found, however, that stratifying cancers by high and low mutation burden changes the correlation of CDK8 expression from shorter to longer survival. CDK8 expression was associated with shorter survival among cancers with below-median mutation burden, not only in AML but also in melanoma, ovarian adenocarcinoma and renal clear cell carcinoma. The observed association of the impact of CDK8 in cancers with low mutation burden is reasonable, since CDK8 regulates transcription and may be expected therefore to have a greater impact in those cancers that are driven primarily by changes in gene expression rather than mutations.

The identification of cancer types where CDK8/CDK19/CCNC are frequently amplified or where their expression correlates with survival suggests a potential role of CDK8/19 in the pathogenesis of such cancers or their treatment response, but it does not automatically mean that such cancers will respond to CDK8/19 inhibitor therapy. Nevertheless, the new correlations identified here provide an obvious impetus for investigating the role of this regulator in the affected types of cancer through detailed pathological analysis and in vivo tumor model studies.

## Figures and Tables

**Table 1:** Correlations of CDK8/CDK19/CCNC expression with patient survival in different types of cancer (OS, overall survival; RFS, relapse-free survival; BCR, biochemical relapse). The correlations for most cancers were derived using Kaplan-Meyer plotter Pan-Cancer RNA-Seq dataset and for a subset of cancers (AML, colon, melanoma, glioblastoma, glioma, prostate) from the available RNA-Seq data as described in Materials and Methods. The hazard ratio (HR) and p values are in black when the gene expression correlates with shorter survival and in green when it correlates with longer survival. *p* values < 0.05 are boldfaced. The p values do not include correction for multiple hypothesis testing.

Cancer Type/Gene			CDK8		CDK19		CCNC	
		n	HR	logrank p	HR	logrank p	HR	logrank p
Acute myeloid leukemia	OS	132	0.63	0.049	0.6	0.026	0.61	0.045
Bladder Carcinoma	OS	405	1.34	0.063	0.71	0.023	1.37	0.042
	RFS	187	1.83	0.091	4.87	0.004	2.68	0.024
Breast cancer	OS	1090	1.61	0.0031	1.43	0.038	1.62	0.01
	RFS	255	1.75	0.0095	1.59	0.032	1.29	0.26
Colon cancer	OS	293	1.34	0.26	0.73	0.21	0.64	0.066
	RFS	109	2.1	0.14	0.59	0.27	0.42	0.079
Cutaneous melanoma	OS	458	1.26	0.093	0.58	0.00048	0.59	0.00046
Esophageal Squamous Cell Carcinoma	OS	81	0.21	0.00053	0.48	0.072	0.53	0.11
	RFS	54	2.57	0.19	1.49	0.42	2.06	0.25
Glioblastoma	OS	152	0.66	0.022	1.28	0.24	0.63	0.019
Head-neck squamous cell carcinoma	OS	500	1.46	0.008	0.6	0.0026	1.53	0.0028
	RFS	124	1.49	0.32	0.43	0.11	2.45	0.016
Kidney renal clear cell carcinoma	OS	530	1.32	0.11	0.49	5.7 × 10^−6^	0.64	0.0028
	RFS	117	0.3	0.017	NA*	0.027	0.16	0.045
Kidney renal papillary cell carcinoma	OS	288	0.68	0.2	2	0.02	1.57	0.15
	RFS	183	1.51	0.38	1.72	0.17	2.18	0.041
Liver hepatocellular carcinoma	OS	71	1.68	0.0059	1.74	0.002	1.25	0.21
	RFS	316	1.38	0.051	2.14	9.1 × 10^−6^	1.28	0.14
Low grade glioma	OS	406	0.43	0.00025	1.43	0.058	1.91	0.0012
	RFS	131	1.33	0.53	0.38	0.034	0.34	0.017
Lung adenocarcinoma	OS	513	1.51	0.0092	0.72	0.07	1.35	0.1
	RFS	300	1.53	0.049	1.34	0.17	0.83	0.38
Lung squamous cell carcinoma	OS	501	1.11	0.44	0.76	0.046	0.86	0.29
	RFS	300	1.49	0.14	2.28	0.015	1.63	0.14
Ovarian cancer	OS	374	1.27	0.072	1.52	0.0015	0.69	0.023
	RFS	177	0.7	0.064	1.49	0.026	0.89	0.54
Pancreatic ductal adenocarcinoma	OS	177	1.27	0.25	1.2	0.4	0.8	0.3
	RFS	69	2.42	0.1	5.31	0.013	1.62	0.25
Pheochromocytoma and Paraganglioma	OS	178	NA*	0.11	13.46	0.0029	4.65	0.052
	RFS	159	7.96	0.033	0.24	0.18	3.18	0.29
Prostate adenocarcinoma	OS	494	4.36	0.019	0.58	0.4	0.48	0.35
	BCR	337	2.46	0.011	2.09	0.039	0.44	0.082
Rectum adenocarcinoma	OS	165	0.59	0.24	0.54	0.12	0.28	0.014
	RFS	47	5.37	0.031	NA*	0.16	0.11	0.027
Sarcoma	OS	259	1.55	0.031	1.41	0.09	1.79	0.0038
	RFS	152	0.71	0.17	1.57	0.074	0.58	0.068
Stomach adenocarcinoma	OS	375	0.55	0.0011	0.81	0.21	0.81	0.21
	RFS	215	0.32	0.00037	1.45	0.3	0.78	0.46
Testicular Germ Cell Tumor	OS	134	0.1	0.015	NA*	0.098	5.09	0.12
	RFS	105	2.37	0.046	0.59	0.19	5.15	0.0029
Thymoma	OS	119	0.28	0.061	0.07	0.00005	0.15	0.0037
Thyroid carcinoma	OS	502	0.67	0.43	0.26	0.055	2.1	0.14
	RFS	353	2.17	0.14	2.19	0.047	0.4	0.12
Uterine corpus endometrial carcinoma	OS	543	1.89	0.0037	1.66	0.023	0.63	0.059
	RFS	422	1.43	0.22	1.88	0.038	1.57	0.1

* number of events too low to compute a statistically valid HR value.
